# Exploring the Choice of Graft Materials in Tympanoplasty: A Perspective on the Use of Temporalis Fascia and Cartilage Grafts. Comment on Ferlito et al. Type 1 Tympanoplasty Outcomes between Cartilage and Temporal Fascia Grafts: A Long-Term Retrospective Study. *J. Clin. Med.* 2022, *11*, 7000

**DOI:** 10.3390/jcm13020543

**Published:** 2024-01-18

**Authors:** Salvatore Ronsivalle, Milena Di Luca

**Affiliations:** ENT Department, Gravina Hospital Caltagirone, ASP Catania, 95041 Caltagirone, Italy; salvatore.ronsivalle@aspct.it

I am writing this letter to discuss and share my thoughts on the recently published research comparing the outcomes of using temporalis fascia and cartilage grafts in type I tympanoplasty [1]. As an experienced otologic surgeon, I have a keen interest in this subject and would like to offer my perspective on the ongoing debate over the choice of graft materials in tympanoplasty.

The authors’ retrospective study of 142 patients who underwent tympanoplasty using either fascia or cartilage grafts is commendable. The paper presents an insightful comparison between the two types of grafts and conclusively shows that both have their merits and demerits. Both the study and the literature highlight how the temporal fascia, given its lower thickness, reports a greater recovery in post-operative hearing compared to the tragal cartilage, which is thicker; on the other hand, the latter presents as a clear advantage, with greater resistance and the lower possibility of re-perforation in the long term compared to the temporal band, which, due to its low thickness, could be re-perforated in the event of complications affecting the middle ear.

Another advantage of tragal cartilage is its use in small perforations or in anterior perforations of tympanic membrane, also skipping the surgical time of temporal fascia harvesting; fascia harvesting also involves a necessary preoperative step, i.e., trichotomy, which is not expected if tragal cartilage is used as a graft.

This aligns with much of the existing literature, which suggests that while fascia offers superior acoustic properties, it is not as durable in the long term. Conversely, cartilage, while more robust, might not offer the same level of immediate functional improvement. Of course, patient-specific conditions and the surgeon’s expertise will always play a part in the final outcome.

One aspect that I believe warrants more attention is the influence of graft thickness on auditory results [2]. The study briefly mentions the use of cartilage grafts, but it would be valuable to delve deeper into the specific thicknesses utilized and their impact on hearing outcomes. For instance, thinner cartilage grafts, around 0.5 mm in thickness, have shown promising results in terms of acoustic benefits, similar to those achieved with fascia grafts. Further investigation into the optimal thickness range for cartilage grafts could provide valuable insights for surgeons when deciding on the graft material.

Additionally, it is worth noting that the tensile strength of fascia decreases with patient age [3]. While the study highlights the long-term stability of cartilage grafts, it would be beneficial to discuss the implications of age-related changes in fascia integrity. As patients age, the tensile strength of fascia diminishes, potentially compromising its durability as a graft material. Considering this factor, cartilage grafts may be a more suitable option for older patients, offering enhanced long-term outcomes and a reduced risk of graft failure or recurrence.

By examining the impact of graft thickness on auditory results and considering the age-related changes in fascia integrity, we can gain a deeper understanding of the factors influencing the choice between temporalis fascia and cartilage grafts in tympanoplasty. This knowledge would empower surgeons to make more informed decisions that optimize both immediate functional improvement and long-term graft stability, ultimately leading to improved patient outcomes and satisfaction.

The choice of graft depends, as already excellently discussed by Ferlito et al., on various factors: one could take into consideration, as a further deciding factor, the functionality test of the Eustachian tube, which is very important. Sato et al. assessed the eustachian tube via the positive and negative pressure equalization tests and clearance tests [4]. The authors stated that the rate of unsuccessful outcomes increased with the degree of tubal dysfunction, indicating that tubal function is closely associated with the outcome of ear surgery. Moreover, Anirban Biswas et al. [5] highlighted how impaired tubal function is the main cause of persistent/recurrent otorrhea and an important contributing factor to the failure of tympanoplasty.

In conclusion, the decision between using temporalis fascia or cartilage in tympanoplasty is complicated, and each has its advantages. The authors have done an excellent job in providing long-term comparative data that will help inform this decision-making process. Ultimately, a balance must be struck between graft integrity and acoustic performance, tailored to each patient’s unique circumstances. I offer my commendations to the authors for their valuable contribution to our understanding of tympanoplasty graft research.
